# Artificial Intelligence-Driven Multi-Omics Approaches in Glioblastoma

**DOI:** 10.3390/ijms26199362

**Published:** 2025-09-25

**Authors:** Giovanna Morello, Valentina La Cognata, Maria Guarnaccia, Giulia Gentile, Sebastiano Cavallaro

**Affiliations:** Institute for Biomedical Research and Innovation, National Research Council (CNR-IRIB), Via P. Gaifami, 18, 95126 Catania, Italy; giovannamariaalessandra.morello@cnr.it (G.M.); valentina.lacognata@cnr.it (V.L.C.); maria.guarnaccia@cnr.it (M.G.); giulia.gentile@cnr.it (G.G.)

**Keywords:** glioblastoma, artificial intelligence, machine learning, deep learning, omics data, molecular classification, personalized medicine

## Abstract

Glioblastoma (GBM) is the most common and aggressive primary brain tumor in adults. It is characterized by a high degree of heterogeneity, meaning that although these tumors may appear morphologically similar, they often exhibit distinct clinical outcomes. By associating specific molecular fingerprints with different clinical behaviors, high-throughput omics technologies (e.g., genomics, transcriptomics, and epigenomics) have significantly advanced our understanding of GBM, particularly of its extensive heterogeneity, by proposing a molecular classification for the implementation of precision medicine. However, due to the vast volume and complexity of data, the integrative analysis of omics data demands substantial computational power for processing, analyzing and interpreting GBM-related data. Artificial intelligence (AI), which mainly includes machine learning (ML) and deep learning (DL) computational approaches, now presents a unique opportunity to infer valuable biological insights from omics data and enhance the clinical management of GBM. In this review, we explored the potential of integrating multi-omics, imaging radiomics and clinical data with AI to uncover different aspects of GBM (molecular profiling, prognosis, and treatment) and improve its clinical management.

## 1. Introduction

Glioblastoma (GBM, formally glioblastoma multiforme) represents the most malignant, aggressive, and common brain tumor in adults [1]. The prognosis for patients with GBM remains poor, with a median survival time of only 15–18 months and a 5-year survival rate of only 5, despite aggressive surgical resection and multi-modality therapy [2,3]. The lack of therapeutic progress may be partly attributed to the inadequate classification of GBM due to its complex cellular and molecular heterogeneity, both within a single tumor and between patients [4]. A deeper functional and molecular characterization of this cancer is thus necessary for improving the understanding of this type of tumor, opening the way to the development of more specific and effective clinical strategies.

Following the advances in high-throughput technologies, in recent decades, large amounts of molecular information have been generated through genomics, transcriptomics and other ‘omics’ profiling, providing a remarkable opportunity to build a more comprehensive molecular landscape of GBM, allowing a better correlation with its diverse phenotypes and offering more reliable prognosis and treatment options for patients [5,6,7]. However, the majority of studies have focused on the analysis of a single dataset or omics domain, while the integration and analysis of different omics layers, together with their integration with other clinical and pathology data, may offer a better picture of GBM etiology, pathogenesis, and treatment options [8,9,10,11,12].

Integrating genotype, phenotype and environmental variables into a unified classification and in a clinically relevant manner represents a challenging task. The application of artificial intelligence (AI) algorithms is now emerging as a powerful tool in overcoming the often-prohibitive complexity of GBM heterogeneity, allowing researchers to retrieve relevant information from the integrative analysis of large and complex multi-omics datasets and, consequently, improving the diagnostic accuracy, prognosis prediction and therapeutic target identification [13,14,15,16,17,18,19,20].

**Figure 1:**
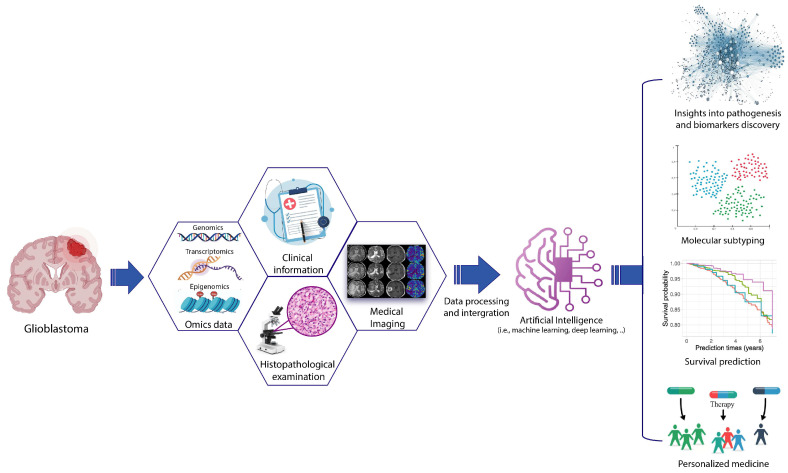
**Applications of multi-modal data integration strategies and artificial intelligence for GBM precision medicine.** This figure illustrates an AI-driven pipeline for personalized GBM interventions that leverages individual patient data to molecularly characterize and subtype GBM samples, predict clinical evolution and treatment sensitivity, and support personalized therapeutic strategies. AI-based data integration tools can combine multiple omics layers (such as genomics, epigenomics, transcriptomics) with clinical, histopathology and medical imaging data, allowing platforms to present a unified view of biological processes, evaluate interrelationships, identify patterns, and detect significant molecular changes across conditions. Beyond providing biological insights, these computational frameworks demonstrate high accuracy and sensitivity in predicting disease outcomes, identifying biomarkers, and supporting tailored treatment strategies through the integration of large-scale omics data.

In this review, we will explore the current applications of AI methods for the analysis and integration of omics and non-omics data (e.g., radiomics, clinical data) GBM data, examining their contribution in obtaining an in-depth understanding of the various molecular mechanisms underlying GBM tumorigenesis and tumor progression, as well as discovering novel biomarkers for a better diagnosis, prognosis and prediction of treatment response (Figure 1).

## 2. Overview of Machine Learning and Artificial Intelligence Models

**Figure 2:**
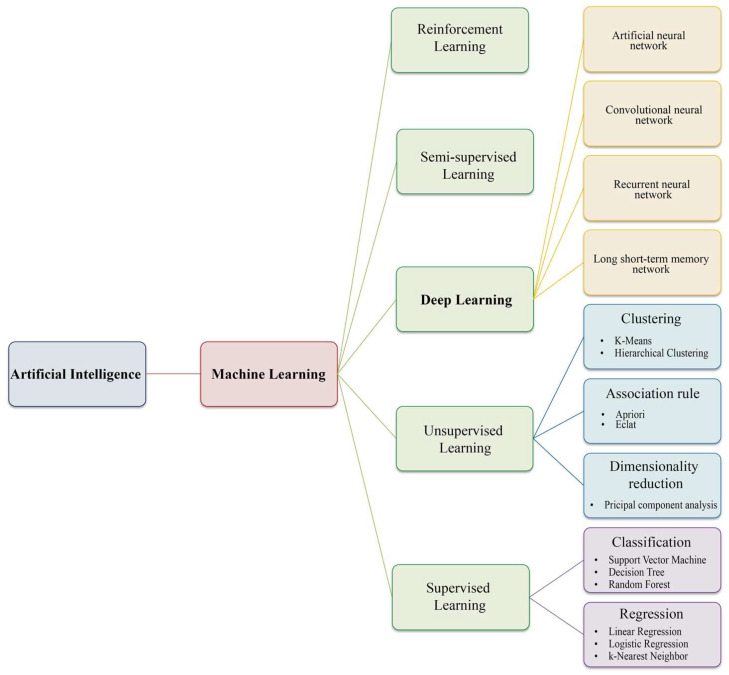
**A hierarchical representation of the most representative artificial intelligence, machine and deep learning algorithms.** Machine Learning (ML) is a main subset of Artificial Intelligence (AI), and its diverse methodologies can be classified into four principal branches: Supervised Learning, Unsupervised Learning, Semi-supervised Learning and Reinforcement Learning. The image above provides a schematic representation of these branches and their associated sub-branches, offering a concise overview of the landscape of ML. Supervised Learning involves classification and regression, where models are trained with labeled data. Unsupervised Learning focuses on clustering, association and dimensionality reduction to find patterns in unlabeled data. Semi-supervised algorithms utilize a combination of both labeled and unlabeled data to enhance ML task performance, as well as reinforcement learning, which improves model performance through interaction with the environment. Deep Learning (DL) is a specific type of ML that uses complex Artificial Neural Networks (ANNs). Each of these branches employs various techniques and algorithms tailored to specific types of data and problem domains.

To effectively introduce the potential of integrating AI into omics-driven GBM studies to a broader audience, it is crucial first to understand the different types of AI and how each uniquely manages and interprets data. Indeed, the field of AI encompasses a range of algorithms and techniques that enable the extraction and processing of high-dimensional data (clinical, biochemical, omics data and medical imaging) as well as their storage, integration, analysis and interpretation [21,22,23] (Figure 2).

The term machine learning (ML) refers to a branch of AI focused on developing algorithms and statistical models, like regression, classification analysis, artificial neural networks and data clustering, which enable computers to analyze input data, identify relationships and patterns between the input features, and make predictions from data without explicit programming [24]. Within this context, the algorithms of feature selection can be categorized into *supervised*, *unsupervised*, *semi-supervised*, *reinforcement learning* and *deep learning* features, depending on the expected output and the input data type [25,26] (Figure 2).

In ***supervised learning***, the training dataset consists of pairs of input data with their corresponding known output labels (e.g., case and control) that are analyzed, producing an inferred function that can be used by the system to “learn” patterns for the subsequent classification of new samples [27,28]. The algorithm is “supervised” by the labeled data that act as a teacher, providing the algorithm with a clear understanding of the relationships between input features and corresponding output variables. Classical, widely used supervised ML techniques, such as *Random Forest*, *Support Vector Machine (SVM)*, *k-Nearest Neighbor*, *Decision Trees*, *Linear* and *Logistic regression*, have been instrumental in omics data analysis and classification [29,30,31] (Figure 2).

In ***unsupervised learning***, the input dataset is represented by unlabeled examples without predefined classes, and the algorithm operates independently to identify hidden patterns or relationships within the unlabeled input data [32,33,34]. Examples of unsupervised learning include (i) *clustering*, (ii) *association* and (iii) *dimensionality reduction* (Figure 2). *Clustering algorithms* (i.e., hierarchical and K-Means clustering) involve grouping similar sets of data based on various criteria, operating on the principle that data points in the same group are more similar to each other than to those in other groups [35,36]. *Association* rule learning deals with uncovering hidden relationships between variables in large datasets that frequently occur together [33]. *Dimensionality reduction* is a technique used to decrease the number of features in a dataset while retaining most of its original information. This approach enables the reduction of data dimensionality while preserving its structure, resulting in a significant speed-up in the time required to train the ML algorithm and, consequently, improving both the performance and results of the analysis [33,37]. Principal component analysis (PCA) is one of the easiest, most intuitive and frequently used unsupervised algorithms for dimensionality reduction, projecting data onto its orthogonal feature subspace, to identify hidden patterns or structures and reveal the most important features in the data that provide the most signal [33,38].

Two other specialized methods often used in cancer diagnostics include ***semi-supervised algorithms***, which utilize a combination of both labeled and unlabeled data to improve ML task performance, and ***reinforcement learning***, a type of ML paradigm where an agent learns to make decisions by interacting with an environment (Figure 2) [39,40,41].

***Deep learning*** (DL) has emerged in recent years as a leading class of ML algorithms, demonstrating improved performance of classifiers, surpassing traditional ML algorithms, principally in the context of high-dimensional data analysis and integration [42,43,44,45]. DL employs artificial neural networks (ANNs), inspired by the simplification of neurons in the brain, which are composed of hidden layers that perform various operations to discover complex representations of the data. ANN consists of densely interconnected artificial neurons, usually arranged into layers: an input layer that collects the input data, which is then processed in the hidden layers used to find any hidden features in the data, while the output layer generates the final expected output [46]. Within each layer, a neuron receives input from neurons in the preceding layer and transmits output to neurons in the subsequent layer. Some of the most fundamental NN-based methods recently applied in omics applications and human pathology are (i) *convolutional neural networks* (CNNs), (ii) *recurrent neural networks* (RNNs) and (iii) *long short-term memory networks* (LSTMs) [47] (Figure 2). The *CNN* is the most famous and commonly employed algorithm for processing image and omics data into a two-dimensional map, demonstrating, among other things, remarkable capabilities in the detection of multiple gene sequence structures, including protein binding sites and enhancer sequences [48,49]. The other two main types of networks, *RNNs* and *LSTMs*, are also used for processing multi-omics data, demonstrating their ability to consistently outperform other DL techniques due to their capacity for processing long-ordered sequences and memorizing long-range information from sequential and time-series data [47]. The aptitude of DL to automatically learn and extract features from raw data has proven invaluable in capturing intricate dependencies within datasets and extracting deeper, more valuable insights from their data. Despite their effectiveness, DL models are very computationally intensive, necessitating high-throughput or high-performance hardware, which has driven the development of more efficient and flexible architectures [50].

The choice of an appropriate AI model is crucial for analyzing and integrating diverse types of omics and non-omics data, as each approach offers unique strengths in uncovering biological insights and profoundly influences the accuracy of outcome prediction, biomarker discovery, and the stratification of patient heterogeneity [42,51,52,53]. Supervised ML algorithms are well-suited when reliable phenotype/outcome data (e.g., disease status, treatment response) are available, as they allow the model to generate predictions with a clearly defined target variable. However, they typically require substantial amounts of well-annotated data for training. Applications of supervised ML in omics include GWAS-based risk classification, prediction of variant pathogenicity, and the inference of disease states or treatment response from methylation and/or gene expression profiles. In contrast, unsupervised methods are better suited for exploring unlabeled omics datasets, where they can uncover hidden structures such as chromatin states, regulatory elements, and disease pathways, as well as identify novel patient clusters based on genetic background, expression patterns, or molecular networks. The primary limitation of unsupervised approaches is the difficulty in evaluating their predictions due to the absence of explicit target labels. While traditional ML techniques have shown partial success in generating predictive models for omics analysis, they often struggle to capture the complex relationships within data required for highly accurate prediction. DL extends these capabilities by integrating high-dimensional and heterogeneous data sources. It is particularly valuable for biological questions that require learning complex, non-linear sequence–structure–function relationships, such as regulatory motif detection, protein folding, and enhancer–promoter interactions, that are difficult or impossible to resolve with conventional methods.

## 3. AI for Omics Data Analysis in GBM

Recent advances in high-throughput technologies have enabled systematic investigation of GBM across multiple biological layers, revealing disease-associated alterations that deepen our understanding of GBM’s complex pathophysiology and improve outcome prediction in diagnosis, prognosis and treatment response in a patient-tailored (personalized) manner [8,9,10,11,12]. However, these platforms generate huge, high-dimensional datasets and their effective analysis and integration require advanced computational power for comprehensive analysis. In this regard, advanced AI methods offer innovative approaches for managing the high dimensionality and heterogeneity of data generated by omics techniques, facilitating their integration and enabling the identification of biomarkers and molecular signatures with the aim to significantly promote the development of diagnostic, prognostic and therapeutic strategies in the precision medicine context [54,55,56,57,58,59].

In the following paragraphs, we will focus on the transformative potential of AI-driven analysis for analyzing and integrating omics data with multi-modal clinical data (e.g., imaging radiomics, pathology, clinical notes) to provide a comprehensive and biologically informed framework for patient stratification and outcome prediction in GBM.

### 3.1. AI-Assisted Genomic Prediction Models for GBM

The genetic profile of GBM is highly heterogeneous, involving mutations in several key genes, including *IDH1/2*, *EGFR*, *PTEN*, and *TP53*, as well as extensive chromosomal instability and complex karyotypes. Currently, the implementation of advanced technologies, including whole-genome sequencing and hardware-accelerated computing, is further expanding the catalog of genetic alterations associated with GBM, enabling the differentiation of different subtypes based on their genomic profiles. The combination of large-scale genomics data and AI methods can assist in GBM diagnosis and prognosis, facilitating the identification of driver mutations and dysregulated functional pathways, and, consequently, improving the molecular characterization and classification of this type of tumor [60].

Numerous supervised ML algorithms have been applied to GBM classification and regression tasks due to their simplicity and interpretability, with Logistic Regression and SVM being the most widely utilized. Some notable early examples include the following: (i) an SVM model developed by Shawon et al., which effectively predicted *IDH1* mutation status in GBM patients, achieving 98.8% accuracy [61]; (ii) an ML-based model designed by Nuechterlein et al. to accurately predict mutated *IDH1/2* along with co-deleted 1p/19q in adult diffuse gliomas, using genome-wide somatic copy number alterations from the Cancer Genome Atlas (TCGA) project [62]; (iii) the combination of multiple ML-based classification algorithms (including SVM, gradient boosting, Neural Network, and Decision Tree) by Al Mamlook et al. to classify GBM samples from the TCGA-GBM dataset, with the Decision Tree algorithm emerging as the most effective classifier, achieving a 99% accuracy [63].

More recently, a ML model has been trained on CNV profiles, gene mutation status, and tumor mutational burden (TMB) of surgical specimens to predict prognostic markers for overall survival (OS) in GBM patients [64]. This model, which also integrates clinical, radiological and molecular parameters, identified *EGFR*, *TP53*, *BRAF*, *POLE*, *PTEN* and *NOTCH3* as the most represented genes in predicting OS [64]. Specifically, mutations in *BRAF* and other genes involved in the PI3K-AKT-mTOR signaling pathway were associated with longer OS, while a higher TMB was associated with shorter OS. Similarly, Felici et al. employed two ML models, SVM and Multi-Layer Perceptron (MLP), to identify relevant genetic predictors associated with an increased risk of GBM [65].

A novel investigative approach now integrates radiogenomics (i.e., an advanced tool for precision oncology that combines imaging and genomic data) with ML models to predict specific genetic mutations in GBM patients [19,66,67,68,69]. In this context, Liang et al. described the application of a SVM model based on multimodal magnetic resonance imaging (MRI) in predicting *IDH1* mutation in GBM patients before surgery, supporting its validity in formulating treatment plans and evaluating prognosis [68]. Another SVM model was used to predict the genetic status and localization of GBM driver genes, such as *EGFR*, *PDGFRA*, and *PTEN*, within GBM using MRI scans [67,70]. Similarly, Akbari et al. employed SVM–based approaches to construct an imaging signature of the most common *EGFR* driver mutation, EGFRvIII, which results from an in-frame deletion that leads to a splice variant and appears to enhance tumor proliferation, invasion, and angiogenesis, through multiple mechanisms, including elevated mitotic rate and reduction in apoptosis [71].

While ML-based algorithms have been the focus of the discussion so far, DL approaches have also been extensively used to examine GBM tumors using genomic data, to develop automatic diagnosis and prognosis systems [8,9,10,11,12]. The recently developed *CHARM* (Cryosection Histopathology Assessment and Review Machine), a DL framework that enables the genomic profiling of brain tumors during surgery, showed the ability to predict key molecular alterations, including the *IDH* mutation and 1p/19q codeletion status, offering real-time guidance on how best to remove or treat the glioma [72]. Tang et al. proposed a multi-task CNN framework for preoperative OS time prediction by simultaneously predicting important genomic biomarkers (*MGMT*, *IDH*, 1p/19q, and *TERT*) for GBM patients, which significantly outperformed conventional radiomics-based ML methods [73]. In addition, another unsupervised DL model was implemented to find hidden structure within GBM genomic data for the purposes of providing insight into disease mechanisms underlying tumor subtypes, which results in effective prediction of OS for patients [74].

### 3.2. Transcriptome-Based AI Approaches for Advanced GBM Diagnosis and Treatment

With the advance of high-throughput omics platforms (i.e., microarrays and RNA-seq) and computational analysis tools, the analysis of the transcriptomic features of GBM has demonstrated high promise for deconvolving the complexity of this type of tumor and identifying previously unrecognized molecular subsets with variations in clinical outcomes, patient survival and treatment response [75,76,77,78,79,80,81]. Transcriptional patterns, in fact, reflect the spatial and temporal diversity during cancer evolution and thus, their analysis is considered a valuable approach for quantitative, reproducible evaluation of inter- and intra-tumoral variations or similarities [82,83,84].

Over the past decades, extensive transcriptomic profiling studies have leveraged large molecular cancer databases (e.g., the TCGA project and the International Cancer Genome Consortium), providing valuable insights into the transcriptional regulation of GBM and defining distinct molecular subgroups associated with prognostic significance [4,7,85,86,87,88,89]. However, it is now clear that GBM subgroups are highly dynamic and can diverge spatially and temporally within a single tumor, complicating the development of effective treatments that target the various mixtures of cell subtypes within the tumor [90].

In this regard, as the computational complexity of omics data analysis grows, the emerging ML-based classifiers are proving to be powerful tools for finding patterns in large high-dimensional gene expression datasets, predicting clinical outcomes and appropriately classifying GBM cases into robust molecular subtypes, with the aim to increase our understanding of disease pathophysiology and enable personalized medical treatments that take into account each patient’s unique biomolecular profile [91,92,93,94,95].

Based on their potential diagnostic utility, the majority of AI-driven transcriptomics studies have focused on the development of classifiers with a small number of genes that accurately classify GBM samples into different subtypes with significantly different clinical outcomes [96,97,98,99,100,101,102,103]. Among these, a modified SVM Recursive Feature Elimination algorithm, in combination with pathway enrichment analysis, was used to develop a 35-gene signature that accurately discriminated between rapidly progressing and slowly progressing GBM patients [104]. Another interesting example is represented by Mao et al., who implemented a deep NN to combine genome expression profiles of GBM samples into unified standardized normal distribution data, which can be used as a feasible tool for accurately classifying GBM subtypes [105]. A recent study supported the clinical utility of a classifier generated by multivariate regression analysis, based on the differential expression of four transcription factors (*LHX2*, *MEOX2*, *SNAI2*, and *ZNF22*), which can be used as independent predictors for the prognosis of patients with GBM [106]. In another recent work, Handayani et al. integrated statistical, ML, and DL approaches to analyze TCGA RNA-Seq gene expression data and clinical data to identify prognostic gene expression biomarkers associated with overall survival in GBM [107]. This DL-based model (named *DeepSurv*) identified 10 key prognostic genes (*CMTR1*, *RPL23AP42*, *TSPYL1*, *AC011287.1*, *RPL7L1P8*, *CCDC107*, *AL354743.2*, *GMPR*, *PPY*, and *MT-TL1*) implicated in processes such as purine metabolism, RNA processing, and neuroendocrine signaling [107]. A further deep multilayer perceptron network was also employed to develop a 39-gene signature, which showed good predictive power in predicting GBM patient survival risk [108]. This model revealed many genes of interest to GBM stem cells’ mechanism and/or treatment resistance, which may be useful in informing patient therapy, including *POSTN*, *TNR*, *BCAN*, *GAD1*, *TMSB15B*, *SCG3*, *PLA2G2A*, *NNMT*, *CHI3L1,* and *ELAVL4* [108]. Similarly, another DL model (named *GBMPurity*) was recently developed for estimating the purity of *IDH*-wild type primary GBM from bulk RNA-seq data, offering valuable insights for guiding further biological investigations [109]. Remarkably, *MTRNR2L12* and *MTRNR2L8*, two isoforms of *MT-RNR2* encoding a peptide with oncogenic effects in GBM, emerged as the most influential genes in purity estimations [109].

In addition to their diagnostic and predictive applications, ML-driven transcriptomic models are also showing promise in predicting GBM treatment outcomes [100,110,111,112]. To this end, Cao et al. developed a 4-gene signature-derived risk score model that could predict the clinical progression status and therapy response in GBM patients, supporting its utility for guiding therapeutic strategies [100]. Jiang et al. supported the clinical utility of a 79-gene AI prognostic signature developed by integrating 10 ML algorithms that showed good performance in accurately predicting patient prognosis and immunotherapy response in GBM [110]. Interestingly, a recent study described a classification model, leveraging gradient boosting with eXtreme Gradient Boosting (XGBoost), showing high accuracy in distinguishing between GBM and osteosarcoma, which originate from the same lineage, also revealing a set of common novel diagnostic and prognostic biomarkers and therapeutic targets with potential clinical implications for improving patient survival in both tumor types [111].

### 3.3. AI Integrates Epigenomic Signatures and Imaging

In the era of big data and AI, a noteworthy perspective for GBM stratification is emerging by training ML algorithms using epigenetic/epigenomic-related data (methylation profiles, lncRNAs and miRNAs signatures) [113]. Multiple research groups are employing this approach, highlighting some interesting characteristics for GBM classification and clinical management.

In a recent work, Jian Shi applied a SVM to classify and predict bevacizumab-responsive GBM patients based on existing miRNA profiling datasets, and reported a panel of differentially expressed miRNAs specifically involved in the stratification of GBM bevacizumab-responsive subtypes [91]. Similarly, Zhang et al., by comparing six different ML algorithms (LassoLR, Boruta, Xgboost, SVM, Random Forest and Pamr), were able to construct and screen a tumor-infiltrating immune cell-associated lncRNAs signature (*TIIClnc*) to reliably distinguish the survival rate of GBM patients within four independent datasets [114]. This *TIIClnc*, obtained by integrating and analyzing the gene expression data of purified immune cells, GBM cell lines or bulk GBM tissues, was revealed to be a functional indicator of immune cell infiltration status in GBM, showing a considerable predictive accuracy for GBM immunotherapy response and prognosis [114].

Alongside the epigenomic changes driven by the class of non-coding RNAs, DNA methylation (DNAm) patterns play a crucial role in brain tumor formation, progression and classification. In this context, ML algorithms are being used to identify GBM-related N6-methyladenosine (m6A) regulatory factors and to detect specific m6A modification patterns (m6Ascore) for predicting GBM patients’ prognosis and immunotherapy response [115].

A particularly significant epigenetic marker is the promoter methylation of O^6^-methylguanine-DNA methyltransferase (*MGMT*), which encodes a DNA repair protein involved in defending cells against mutagenesis and the toxicity of alkylating agents [116]. *MGMT* promoter hypermethylation, or *MGMT* gene silencing, represents a key predictive biomarker for patient response to first-line temozolomide chemotherapy, with hypermethylation generally associated with a better response [116]. Currently, the identification of the *MGMT* promoter methylation state is obtained after biopsy and genetic testing, which represents an invasive operation, often limited to the easily accessible areas of the tumor. It is costly and time-consuming, often delaying important therapeutic decisions [117]. However, recent studies are encouraging the use of AI models to predict the *MGMT* promoter status from the MRI scans of the tumor, by expanding a new field of research (radiomics), whose goal is to extract information from medical images to quantify the brain tumor phenotype [118]. For this aim, traditional ML methods (such as Random Forest and SVM) have been introduced, reporting different prediction performances. For example, Xi et al. trained a SVM-based approach on features from T1-weighted image (T1WI), T2-weighted image (T2WI), and enhanced T1WI, and described the best performance when using a combination of all image features [119]. In a different study, 7000 radiomics features were extracted from 82 GBM patients and twelve classifiers (Adaptive boost, Bagging, Naïve Bayes, Decision Tree, Gaussian Naïve Bayes, K-nearest neighbors, Logistic regression, Multilayer perceptron, Quadratic discriminant analysis, Random Forest, Stochastic gradient descent, SVM) were implemented and compared [120]. Among them, the Decision Tree with Select from Model feature selector and LOG (Laplacian of Gaussian) filter in the edema region was the one with the highest performance. Moreover, the XGBoost model has been successfully employed for identifying *MGMT* promoter methylation status in GBM patients with *IDH1* wildtype by analyzing nine highly predicting radiomics features [121], or in a hybrid conformation model with a genetic algorithm (GA)-based wrapper, achieving a sensitivity of 89%, a specificity of 96%, and an accuracy of 92% [122].

Several attempts have been made to classify *MGMT* promoter methylation status using DL solutions. Some of these studies demonstrated the ability of DL models in predicting *MGMT* promoter methylation [117,123,124,125], while in other works, authors found a poor correlation between the imaging data and *MGMT* methylation status, underscoring the importance of further validations to verify the value of these DL approaches [126,127,128,129,130].

A recent interesting work has exploited both ML and DL methods for classifying *MGMT* methylated/unmethylated GBM [131]. SVM, K-Nearest Neighbors, random forest, LightGBM and XGBoost were used to investigate the hand-crafted radiomics features extracted from GBM’s subregions, such as edema, tumor core, and enhancing tumor, while for tissue-level analysis, a deep residual neural network (ResNet-18) with 3D architecture, followed by an EfficientNet-based investigation, was implemented. The authors concluded that the investigated solution yielded successful results, eliminating the need for a complex tumor segmentation step [131].

### 3.4. AI-Based Multi-Modal Integration in GBM

The crucial events driving GBM malignant transformation require multiple genetic and molecular alterations. While, as we discussed above, the single-layer-omics approaches are attempting to untangle mechanisms underlying the onset and progression of GBM, providing researchers with a refinement of disease diagnosis, prognosis, and therapy, they often fail to establish causal associations between molecular changes and clinical manifestations and provide limited insights into the heterogeneity of GBM. To this end, the combined and integrative analysis of multi-omics data with other high-dimensional data (i.e., imaging radiomics, histopathologic and clinical data) as well as the implementation of advanced computational methods are essential to comprehend how the molecular alterations at different but mutually linked layers of genomic regulation contribute to disease formation, define new molecular classification systems, and explore novel diagnostic, prognostic, and therapeutic options for patients [10,11,132,133].

Recent years have seen the development of numerous strategies for integrating multi-modal data, which can be categorized into three main approaches [38,134,135,136,137,138,139,140,141]. Early integration merges all data into a single input matrix. Intermediate integration utilizes models, such as autoencoders, to learn aligned, shared representations that preserve omics-specific signals while capturing cross-omics interactions. Late integration trains separate models on each dataset and combines their predictions. Emerging deep learning frameworks increasingly use hybrid integration, leveraging cross-modal attention and graph networks to combine these strategies, thus integrating information at multiple levels and preventing information loss [142].

The integration of large-scale, high-dimensional data in glioblastoma (GBM) using AI techniques shows significant promise for enhancing molecular classification, defining prognostic risk signatures, and informing precision treatment strategies [98,132,143,144,145,146,147,148,149,150,151,152,153,154,155]. Among these, Lu et al. published a comprehensive multi-omics classifier for GBM molecular subtypes, leveraging six types of molecular data from TCGA GBM samples, including genetic variants, gene expression data, CNV and miRNA expression data and DNA methylation status, that are jointly combined with clinical data and phenotypic inputs to predict GBM patients’ survival time [143]. Recently, a novel integrative transcriptomic and epigenomic classifier, called *iGlioSub*, has been developed, demonstrating a valuable potential for GBM subtype classification [144]. In particular, this classifier utilizes features from both gene expression-based and DNAm-profiles from 304 GBM patients deposited in the TCGA, the Human Glioblastoma Cell Culture resource (HGCC) and other publicly available databases to stratify GBM patients based on DNAm patterns and activated gene pathways [144]. In addition to achieving a classification efficiency greater than other ML-based strategies for classifying GBM subtypes (90% accuracy), *iGlioSub* has demonstrated potential suitability for routine pathology laboratories due to the possibility of implementing it with low-complexity techniques, such as qPCR or pyrosequencing [144].

Some further approaches to GBM classification based on integrated data from both high-throughput gene expression and methylation profiles relied on DL. Munquad et al. developed a biologically interpretable and highly efficient DL framework based on a combination of LASSO (least absolute shrinkage and selection operator) feature selection and a convolutional NN to classify GBM subtypes with high accuracy [148]. More recently, the same authors described *DeepAutoGlioma*, a deep autoencoder and deep neural network-based clinically relevant framework that enables the combination of gene expression data with methylome data to classify glioma subtypes with increased accuracy. They implemented two DL algorithms, i.e., artificial NN and convolutional NN, and evaluated their performance for low-grade gliomas and GBM classification, demonstrating how the integration of transcriptomics and epigenomics data can aid in developing effective diagnostic tools [147].

A novel CNN model, called *PathCNN,* was constructed using integrated multi-omics data of GBM, demonstrating promising predictive performance in differentiating between long-term survival (LTS) and non-LTS, and enabling the identification of plausible pathways associated with survival in glioblastoma [156]. Another robust GBM classifier was developed by Chen et al. by using ML to integrate multiple omics data (CNV, mRNA, and mutation profiles), showing an excellent ability to classify patients with high-risk or low-risk of poor prognosis, and identifying therapeutic agents (NVPBEZ235, GDC0980, dasatinib and XL765) with a subclass-specific efficacy, thus providing a valid preclinical platform for discovering subtype-specific agents for GBM and improving precision treatment strategies [98]. Recently, Xu et al. combined transcriptome, proteomics, and single-cell sequencing data by employing four different ML algorithms, identifying 17 key genes associated with patient prognosis [151]. Hao et al. collected mRNA expression, DNA methylation and microRNA expression data for four cancers, including GBM, and proposed a DL approach (accuracy: 86%) for cancer survival prediction [157]. Similarly, the deep neural network, *DeepSigSurvNet*, integrated gene expression and copy number data from a set of key genes and signaling pathways to model patients’ survival in four types of cancer, including GBM [158].

With the intent of enhancing the robustness of molecular classifiers and facilitating their translation into interpretable clinical decisions and robust clinical assays, recent studies have sought to evaluate the potential of predictive models that integrate multi-omics data with medical imaging data (i.e., radiomics) and other clinical features [145,146,159]. For example, the *Bio-ATT-CNN*, a CNN based on an attention mechanism, was implemented to predict long-term survival in GBM patients by combining radiomic feature values with mRNA expression, CNV, and DNA methylation data across different molecular subtypes of GBM [160]. Chaddad et al. used a random forest model to classify GBM patients into groups corresponding to short and long-term survival by integrating radiomic features with genomics, proteomics, or methylation status [161]. By combining SVM and DL methods, Kazerooni et al. assessed the potential of integrating multi-omics prognostic characteristics, including clinical measures, radiomics, *MGMT* methylation, and genomics, to predict OS in GBM patients, revealing two genes, *RB1* and *NOTCH2*, previously studied for their role in oncogenesis, as having significant associations with survival [145].

Importantly, multi-modal integrative frameworks can also help to resolve intra-tumoral heterogeneity in GBM at high resolution, by uncovering latent resistance drivers or subclonal architectures that remain undetectable in single-layer datasets [90,162,163]. Integrative analysis, combining single-cell and spatial omics with radiomic imaging features, enables AI models to resolve intra-tumoral heterogeneity in GBM at high resolution, allowing for non-invasive mapping of the molecular diversity across tumor regions, identifying region-specific pathways or immunogenic targets and contributing to explain why different tumor subregions respond differently to therapy [164,165]. Models trained on multi-regional biopsy and imaging data can further map the spatial distribution of key mutations, while clustering analysis reveals subpopulations that may drive tumor progression or therapeutic failure. Beyond improving our understanding of GBM biological heterogeneity, AI has also yielded predictive frameworks linking heterogeneous tumor features to clinical outcomes, including survival, recurrence, and therapeutic resistance. For instance, Lam et al. employed an XGBoost model that integrated transcriptional and proteomic data with patient-derived clinical information, uncovering a regionally variable MYC-KRAS molecular axis that cooperates with hypoxia to generate a three-dimensional model of intra-tumoral heterogeneity, potentially guiding the design of drug combination strategies to overcome GBM therapy resistance [165]. More recently, the multi-center initiative called MOSAIC (Multi-Omics Spatial Atlas in Cancer) was launched to systematically profile thousands of cancer samples by integrating spatial and single-cell data with complementary modalities through AI and other computational approaches, with the primary goal of advancing precision oncology through a comprehensive characterization of cancer heterogeneity [166].

Overall, the proper integration of multi-modal measures by ML and DL has demonstrated the ability to produce robust GBM signature score models with superior performance and high application value in predicting the survival of GBM patients, providing important steps towards the development of a personalized clinical management of GBM [145,146].

## 4. Challenges and Future Perspectives

As extensively discussed, GBM is a highly complex and heterogeneous disease, and integration of multi-modal high-dimensional data using AI-driven tools has become indispensable for understanding its underlying molecular mechanisms, identifying tumor-specific molecular drivers and therapeutic targets, and ultimately implementing personalized medicine approaches. Nonetheless, translating these complex findings into actionable clinical strategies remains a challenge. Overcoming this hurdle requires the development of standardized analytical pipelines and prospective clinical trials to test AI-guided treatment decisions [38,56,167].

The generalizability and interpretability of AI models remain two significant barriers to their widespread clinical adaptation. In fact, the reliability of AI models depends on the integrity and completeness of clinical and biomedical data, which necessitates rigorous quality control and validation protocols. The growing complexity of AI models requires substantial computational resources, which may not be accessible to all research institutions and many AI-driven approaches, particularly DL models, operate as “black boxes,” making it difficult for clinicians to understand how predictions are generated, especially when analyzing complex omics datasets and drug-response mechanisms [168]. In addition, to date, most AI applications in GBM and oncology have been trained on relatively small and medium-scale patient populations, and even large-scale resources like TCGA provide sparse clinical drug response information [169]. The multi-institutional nature of most clinical trials for GBM, as well as inconsistencies in data annotation, missing values, and variability in high-dimensional biomedical data across different institutions, further complicates the acquisition of uniform datasets, thereby affecting the reliability and reproducibility of AI predictions. Thus, the implementation of large, standardized and annotated datasets from multiple institutions with clinical, neuroimaging, neuropathologic and molecular multi-omics data that cover diverse patient populations, as well as their nested cross-validation and independent external replication, are needed to address these gaps and develop accurate AI models, ensuring their successful translation into clinical applications [168]. In this regard, a range of preprocessing techniques, including data normalization methods (i.e., log transformation, quantile normalization, and z-score scaling) and batch effect correction tools (i.e., ComBat, Harmony, and Limma), has been adopted to harmonize data collected from different platforms [170,171]. In addition, community-driven initiatives have enhanced consistency, accessibility, and interoperability within the multi-omics research ecosystem. Among these, the National Cancer Institute Genomic Data Commons (GDC) is a notable example of a data repository that uniformly processes multi-omics profiles and associated clinical data, enabling data sharing and collaborative analysis in support of precision medicine in oncology [172].

The clinical implementation of AI-driven models in GBM care also introduces ethical considerations due to the vast amounts of personal data they process, including patient data privacy, information security, and potential algorithmic biases [173,174,175]. Without adequate safeguards and well-defined guidelines for the development, validation, and approval of AI tools in healthcare, these systems may enable unauthorized access, misinformation, re-identification of anonymized data, biased algorithmic outcomes, and a lack of transparency in AI decision-making processes. Furthermore, providing expensive precision therapies to patients with limited access to healthcare resources poses additional ethical challenges. Ensuring that these technologies are equitably accessible is, in fact, crucial to prevent widening disparities between healthcare systems worldwide.

Addressing technical and ethical challenges is crucial for the effective implementation of AI in GBM management. By ensuring transparent methodologies, multidisciplinary collaboration, and inclusivity, AI implementation can be optimized to improve patient outcomes and advance the field of neuro-oncology.

## 5. Conclusions

GBM is a highly heterogeneous cancer characterized by multiple genetic and molecular alterations. Despite significant advances, it is still a deadly disease with an extremely poor prognosis. AI and ML are increasingly emerging as major breakthroughs and pioneering approaches for enhancing GBM knowledge, as well as for establishing defined subgroups, developing early diagnostic methods, and personalized treatment strategies. Although the successful translation of AI-integrated approaches into GBM management requires addressing challenges and considerations specific to the medical domain (i.e., scarcity of patient data, the lack of standardization in omics data processing, the lack of standards, and imbalance in the data used to build the model), current research efforts are resolving the significant shortcomings, providing very promising applications for implementing research findings in clinical practice. The increasing availability of larger, high-quality omics datasets, medical images, and phenotypic data, together with the development of more robust and accurate algorithms that combine and integrate all these data, are fueling the promising applications of AI technology in the clinical management of GBM, making them pivotal in achieving the final goal of a single unified classification of GBM heterogeneity as well as leading towards the development of personalized healthcare.
